# *N*′-(2-Chloro­benzyl­idene)-2-hydr­oxy-3-methyl­benzohydrazide

**DOI:** 10.1107/S1600536810012110

**Published:** 2010-04-02

**Authors:** You-Yue Han, Qiu-Rong Zhao

**Affiliations:** a Department of Chemistry and Life Science, Chuzhou University, Chuzhou, Anhui 239000, People’s Republic of China

## Abstract

In the title compound, C_15_H_13_ClN_2_O_2_, the dihedral angle between the two benzene rings is 3.4 (5)° and the mol­ecule adopts an *E* configuration with respect to the C=N bond. There is an intra­molecular O—H⋯O hydrogen bond in the mol­ecule, which generates an *S*(6) loop. In the crystal structure, mol­ecules are linked through inter­molecular N—H⋯O hydrogen bonds, forming *C*(4) chains running along the *a* axis.

## Related literature

For the biological properties of hydrazone compounds, see: Patil *et al.* (2010 ▶); Cukurovali *et al.* (2006 ▶). For related structures, see: Mohd Lair *et al.* (2009 ▶); Lin & Sang (2009 ▶); Suleiman Gwaram *et al.* (2010 ▶); Li & Ban (2009 ▶); Lo & Ng (2009 ▶); Ning & Xu (2009 ▶); Zhu *et al.* (2009 ▶). For reference structural data, see: Allen *et al.* (1987 ▶).
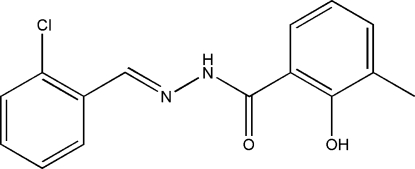


## Experimental

### 

#### Crystal data

C_15_H_13_ClN_2_O_2_
*M* *_r_* = 288.72
Monoclinic, 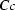

*a* = 7.084 (2) Å
*b* = 27.010 (3) Å
*c* = 7.755 (2) Å
β = 111.229 (3)°
*V* = 1383.1 (6) Å^3^
*Z* = 4
Mo *K*α radiation
μ = 0.28 mm^−1^
*T* = 298 K
0.12 × 0.10 × 0.10 mm

#### Data collection

Bruker SMART CCD diffractometer
Absorption correction: multi-scan (*SADABS*; Bruker, 2001 ▶) *T*_min_ = 0.967, *T*_max_ = 0.973
3856 measured reflections
1981 independent reflections
1145 reflections with *I* > 2σ(*I*)
*R*_int_ = 0.151

#### Refinement

*R*[*F* ^2^ > 2σ(*F* ^2^)] = 0.083
*wR*(*F* ^2^) = 0.220
*S* = 0.92
1981 reflections
183 parameters
2 restraints
H-atom parameters constrained
Δρ_max_ = 0.39 e Å^−3^
Δρ_min_ = −0.45 e Å^−3^
Absolute structure: Flack (1983 ▶), 470 Friedel pairs
Flack parameter: 0.29 (17)

### 

Data collection: *SMART* (Bruker, 2007 ▶); cell refinement: *SAINT* (Bruker, 2007 ▶); data reduction: *SAINT*; program(s) used to solve structure: *SHELXTL* (Sheldrick, 2008 ▶); program(s) used to refine structure: *SHELXTL*; molecular graphics: *SHELXTL*; software used to prepare material for publication: *SHELXTL*.

## Supplementary Material

Crystal structure: contains datablocks global, I. DOI: 10.1107/S1600536810012110/hb5388sup1.cif

Structure factors: contains datablocks I. DOI: 10.1107/S1600536810012110/hb5388Isup2.hkl

Additional supplementary materials:  crystallographic information; 3D view; checkCIF report

## Figures and Tables

**Table 1:** Hydrogen-bond geometry (Å, °)

*D*—H⋯*A*	*D*—H	H⋯*A*	*D*⋯*A*	*D*—H⋯*A*
N1—H1⋯O1^i^	0.86	2.41	3.202 (7)	154
O2—H2⋯O1	0.82	1.92	2.641 (7)	146

Symmetry code: (i) 
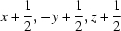
.

**Fig. 1.**
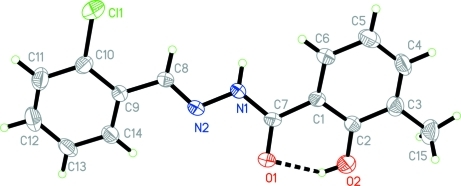
The molecular structure of (I) with 30% probability displacement ellipsoids for non-H atoms. Intramolecular O–H···O hydrogen bond is shown as a dashed line.

**Fig. 2.**
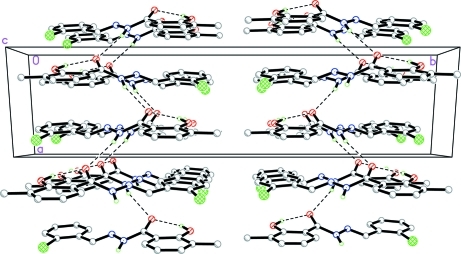
The packing of (I) viewed along the c axis. Hydrogen bonds are shown as dashed lines.

## supplementary crystallographic information

### Comment

Hydrazone compounds have been widely investigated for their biological properties (Patil *et al.*, 2010; Cukurovali *et al.*, 2006). Furthermore, the crystal structures of the hydrazone compounds have also attracted much attention in recent years (Mohd Lair *et al.*, 2009; Lin & Sang, 2009; Suleiman Gwaram *et al.*, 2010). In the present work, the title new hydrazone compound is reported.

In the molecule of the title compound, Fig. 1, the dihedral angle between the two benzene rings is 3.4 (5)°. The molecule adopts an *E* configuration with respect to the C═N bond. There is an intramolecular O–H···O hydrogen bond (Table 1) in the molecule. All the bond lengths are within normal ranges (Allen *et al.*, 1987), and are comparable with those in the similar compounds (Li & Ban, 2009; Lo & Ng, 2009; Ning & Xu, 2009; Zhu *et al.*, 2009).

In the crystal structure, molecules are linked through intermolecular N–H···O hydrogen bonds (Table 1) to form chains running along the *a* axis (Fig. 2).

### Experimental

A mixture of 2-chlorobenzaldehyde (0.140 g, 1 mmol) and 2-hydroxy-3-methylbenzohydrazide (0.166 g, 1 mmol) in 50 ml me thanol was stirred at room temperature for 1 h. The mixture was filtered to remove impurities, and then left at room temperature. After a few days, colourless blocks of (I) were formed.

### Refinement

H atoms were positioned geometrically and refined using the riding-model approximation, with C–H = 0.93 or 0.96 Å, O–H = 0.82 Å, N–H = 0.86 Å, and U_iso_(H) = 1.2U_eq_(C,*N*) or U_iso_(H) = 1.5U_eq_(methyl C and O).

### Crystal data

**Table:**

C_15_H_13_ClN_2_O_2_	*F*(000) = 600
*M**_r_* = 288.72	*D*_x_ = 1.387 Mg m^−^^3^
Monoclinic, *C**c*	Mo *K*α radiation, λ = 0.71073 Å
Hall symbol: C -2yc	Cell parameters from 975 reflections
*a* = 7.084 (2) Å	θ = 2.6–24.5°
*b* = 27.010 (3) Å	µ = 0.28 mm^−^^1^
*c* = 7.755 (2) Å	*T* = 298 K
β = 111.229 (3)°	Block, colorless
*V* = 1383.1 (6) Å^3^	0.12 × 0.10 × 0.10 mm
*Z* = 4	

### Data collection

**Table:**

Bruker SMART CCD diffractometer	1981 independent reflections
Radiation source: fine-focus sealed tube	1145 reflections with *I* > 2σ(*I*)
graphite	*R*_int_ = 0.151
ω scans	θ_max_ = 27.0°, θ_min_ = 3.0°
Absorption correction: multi-scan (*SADABS*; Bruker, 2001)	*h* = −4→9
*T*_min_ = 0.967, *T*_max_ = 0.973	*k* = −34→34
3856 measured reflections	*l* = −9→9

### Refinement

**Table:**

Refinement on *F*^2^	Secondary atom site location: difference Fourier map
Least-squares matrix: full	Hydrogen site location: inferred from neighbouring sites
*R*[*F*^2^ > 2σ(*F*^2^)] = 0.083	H-atom parameters constrained
*wR*(*F*^2^) = 0.220	*w* = 1/[σ^2^(*F*_o_^2^) + (0.1271*P*)^2^] where *P* = (*F*_o_^2^ + 2*F*_c_^2^)/3
*S* = 0.92	(Δ/σ)_max_ = 0.001
1981 reflections	Δρ_max_ = 0.39 e Å^−^^3^
183 parameters	Δρ_min_ = −0.45 e Å^−^^3^
2 restraints	Absolute structure: Flack (1983), 470 Fridel pairs
Primary atom site location: structure-invariant direct methods	Flack parameter: 0.29 (17)

### Special details

**Table:**

Geometry. All esds (except the esd in the dihedral angle between two l.s. planes) are estimated using the full covariance matrix. The cell esds are taken into account individually in the estimation of esds in distances, angles and torsion angles; correlations between esds in cell parameters are only used when they are defined by crystal symmetry. An approximate (isotropic) treatment of cell esds is used for estimating esds involving l.s. planes.
Refinement. Refinement of *F*^2^ against ALL reflections. The weighted *R*-factor wR and goodness of fit *S* are based on *F*^2^, conventional *R*-factors *R* are based on *F*, with *F* set to zero for negative *F*^2^. The threshold expression of *F*^2^ > σ(*F*^2^) is used only for calculating *R*-factors(gt) etc. and is not relevant to the choice of reflections for refinement. *R*-factors based on *F*^2^ are statistically about twice as large as those based on *F*, and *R*- factors based on ALL data will be even larger.

### Fractional atomic coordinates and isotropic or equivalent isotropic displacement parameters (Å^2^)

**Table:**

	*x*	*y*	*z*	*U*_iso_*/*U*_eq_	
Cl1	0.3375 (3)	0.43050 (6)	0.8129 (3)	0.0785 (7)	
N1	0.2710 (8)	0.24748 (18)	0.8198 (7)	0.0526 (14)	
H1	0.3490	0.2572	0.9277	0.063*	
N2	0.2275 (8)	0.2780 (2)	0.6677 (6)	0.0503 (13)	
O1	0.0826 (8)	0.18614 (19)	0.6388 (6)	0.0704 (15)	
O2	0.1832 (8)	0.09660 (18)	0.7787 (7)	0.0666 (14)	
H2	0.1443	0.1174	0.6964	0.100*	
C1	0.2311 (9)	0.1713 (2)	0.9627 (8)	0.0462 (15)	
C2	0.2276 (10)	0.1188 (2)	0.9446 (8)	0.0505 (16)	
C3	0.2680 (10)	0.0889 (3)	1.1021 (10)	0.0599 (19)	
C4	0.3049 (11)	0.1111 (3)	1.2688 (9)	0.065 (2)	
H4	0.3328	0.0912	1.3730	0.078*	
C5	0.3026 (12)	0.1623 (3)	1.2901 (11)	0.067 (2)	
H5	0.3253	0.1762	1.4055	0.081*	
C6	0.2661 (9)	0.1919 (3)	1.1361 (8)	0.0550 (17)	
H6	0.2648	0.2262	1.1484	0.066*	
C7	0.1867 (9)	0.2013 (2)	0.7943 (8)	0.0489 (16)	
C8	0.2946 (10)	0.3218 (3)	0.7035 (9)	0.0485 (15)	
H8	0.3665	0.3312	0.8251	0.058*	
C9	0.2591 (9)	0.3575 (2)	0.5540 (8)	0.0445 (14)	
C10	0.2758 (10)	0.4085 (2)	0.5897 (9)	0.0548 (18)	
C11	0.2424 (13)	0.4419 (3)	0.4451 (12)	0.070 (2)	
H11	0.2509	0.4757	0.4689	0.084*	
C12	0.1983 (14)	0.4256 (3)	0.2722 (13)	0.078 (2)	
H12	0.1778	0.4485	0.1775	0.093*	
C13	0.1823 (11)	0.3753 (3)	0.2297 (9)	0.066 (2)	
H13	0.1523	0.3645	0.1087	0.079*	
C14	0.2122 (10)	0.3417 (3)	0.3723 (9)	0.0555 (18)	
H14	0.2008	0.3080	0.3462	0.067*	
C15	0.2730 (15)	0.0336 (3)	1.0799 (14)	0.085 (3)	
H15A	0.3750	0.0251	1.0306	0.127*	
H15B	0.1433	0.0224	0.9969	0.127*	
H15C	0.3037	0.0180	1.1982	0.127*	

### Atomic displacement parameters (Å^2^)

**Table:**

	*U*^11^	*U*^22^	*U*^33^	*U*^12^	*U*^13^	*U*^23^
Cl1	0.1018 (16)	0.0557 (10)	0.0756 (12)	−0.0051 (12)	0.0294 (10)	−0.0184 (10)
N1	0.063 (3)	0.044 (3)	0.035 (2)	−0.005 (3)	−0.001 (2)	0.005 (2)
N2	0.056 (3)	0.049 (3)	0.034 (2)	−0.002 (3)	0.002 (2)	0.002 (2)
O1	0.100 (4)	0.057 (3)	0.034 (2)	−0.024 (3)	0.001 (2)	−0.001 (2)
O2	0.087 (4)	0.049 (3)	0.058 (3)	−0.002 (3)	0.020 (3)	−0.005 (2)
C1	0.042 (3)	0.046 (3)	0.039 (3)	−0.001 (3)	0.001 (3)	0.001 (3)
C2	0.050 (4)	0.049 (3)	0.043 (3)	−0.003 (3)	0.005 (3)	0.006 (3)
C3	0.052 (4)	0.056 (4)	0.062 (5)	0.001 (3)	0.009 (3)	0.015 (3)
C4	0.063 (5)	0.075 (5)	0.050 (4)	−0.001 (4)	0.012 (4)	0.025 (4)
C5	0.066 (5)	0.089 (6)	0.043 (3)	−0.008 (4)	0.015 (3)	−0.003 (4)
C6	0.059 (4)	0.059 (4)	0.038 (3)	−0.007 (3)	0.007 (3)	0.001 (3)
C7	0.051 (4)	0.049 (3)	0.034 (3)	−0.005 (3)	0.001 (3)	−0.003 (3)
C8	0.048 (4)	0.049 (4)	0.039 (3)	−0.007 (3)	0.003 (2)	0.001 (3)
C9	0.046 (3)	0.043 (3)	0.041 (3)	0.002 (3)	0.011 (3)	0.003 (3)
C10	0.058 (4)	0.044 (3)	0.060 (4)	−0.003 (3)	0.018 (3)	−0.005 (3)
C11	0.078 (5)	0.047 (4)	0.082 (6)	0.009 (4)	0.024 (4)	0.012 (4)
C12	0.085 (6)	0.071 (5)	0.074 (5)	0.010 (5)	0.024 (4)	0.032 (5)
C13	0.066 (5)	0.082 (5)	0.038 (3)	0.001 (4)	0.006 (3)	0.012 (3)
C14	0.051 (4)	0.061 (4)	0.048 (4)	−0.004 (3)	0.011 (3)	0.008 (3)
C15	0.093 (6)	0.057 (4)	0.100 (7)	0.006 (5)	0.028 (5)	0.024 (5)

### Geometric parameters (Å, °)

**Table:**

Cl1—C10	1.730 (7)	C5—H5	0.9300
N1—C7	1.365 (8)	C6—H6	0.9300
N1—N2	1.379 (7)	C8—C9	1.458 (9)
N1—H1	0.8600	C8—H8	0.9300
N2—C8	1.268 (8)	C9—C14	1.392 (10)
O1—C7	1.234 (7)	C9—C10	1.400 (8)
O2—C2	1.350 (8)	C10—C11	1.392 (10)
O2—H2	0.8200	C11—C12	1.336 (12)
C1—C6	1.392 (9)	C11—H11	0.9300
C1—C2	1.423 (8)	C12—C13	1.392 (12)
C1—C7	1.472 (9)	C12—H12	0.9300
C2—C3	1.405 (9)	C13—C14	1.387 (10)
C3—C4	1.362 (10)	C13—H13	0.9300
C3—C15	1.506 (12)	C14—H14	0.9300
C4—C5	1.394 (11)	C15—H15A	0.9600
C4—H4	0.9300	C15—H15B	0.9600
C5—C6	1.382 (10)	C15—H15C	0.9600
			
C7—N1—N2	118.1 (4)	N2—C8—H8	120.0
C7—N1—H1	121.0	C9—C8—H8	120.0
N2—N1—H1	121.0	C14—C9—C10	118.3 (6)
C8—N2—N1	114.9 (5)	C14—C9—C8	120.7 (6)
C2—O2—H2	109.5	C10—C9—C8	121.0 (6)
C6—C1—C2	119.0 (6)	C11—C10—C9	120.0 (6)
C6—C1—C7	122.8 (6)	C11—C10—Cl1	119.4 (5)
C2—C1—C7	118.1 (5)	C9—C10—Cl1	120.6 (5)
O2—C2—C3	118.4 (6)	C12—C11—C10	120.3 (7)
O2—C2—C1	121.8 (5)	C12—C11—H11	119.8
C3—C2—C1	119.8 (6)	C10—C11—H11	119.8
C4—C3—C2	118.7 (7)	C11—C12—C13	121.9 (7)
C4—C3—C15	122.7 (7)	C11—C12—H12	119.1
C2—C3—C15	118.5 (7)	C13—C12—H12	119.1
C3—C4—C5	122.9 (7)	C14—C13—C12	118.3 (7)
C3—C4—H4	118.6	C14—C13—H13	120.9
C5—C4—H4	118.6	C12—C13—H13	120.9
C6—C5—C4	118.6 (7)	C13—C14—C9	121.2 (7)
C6—C5—H5	120.7	C13—C14—H14	119.4
C4—C5—H5	120.7	C9—C14—H14	119.4
C5—C6—C1	121.0 (7)	C3—C15—H15A	109.5
C5—C6—H6	119.5	C3—C15—H15B	109.5
C1—C6—H6	119.5	H15A—C15—H15B	109.5
O1—C7—N1	121.4 (6)	C3—C15—H15C	109.5
O1—C7—C1	122.9 (6)	H15A—C15—H15C	109.5
N1—C7—C1	115.7 (5)	H15B—C15—H15C	109.5
N2—C8—C9	120.0 (6)		

### Hydrogen-bond geometry (Å, °)

**Table:**

*D*—H···*A*	*D*—H	H···*A*	*D*···*A*	*D*—H···*A*
N1—H1···O1^i^	0.86	2.41	3.202 (7)	154
O2—H2···O1	0.82	1.92	2.641 (7)	146

Symmetry codes: (i) *x*+1/2, −*y*+1/2, *z*+1/2.
